# Autologous transplantation of cytokine-induced killer cells as an adjuvant therapy for hepatocellular carcinoma in Asia: an update meta-analysis and systematic review

**DOI:** 10.18632/oncotarget.15454

**Published:** 2017-02-17

**Authors:** Xiu-Rong Cai, Xing Li, Jin-Xiang Lin, Tian-Tian Wang, Min Dong, Zhan-Hong Chen, Chang-Chang Jia, Ying-Fen Hong, Qu Lin, Xiang-Yuan Wu

**Affiliations:** ^1^ Department of Medical Oncology, the Third Affiliated Hospital of Sun Yat-sen University, Guangzhou 510630, People's Republic of China; ^2^ Guangdong provincial Key Laboratory of Liver Disease Research, Guangzhou 510630, People's Republic of China; ^3^ Cell-gene Therapy Translational Medicine Research Center, the Third Affiliated Hospital of Sun Yat-sen University, Guangzhou 510630, People's Republic of China

**Keywords:** cytokine induced killer cell, liver cancer, adoptive cells therapy, immunotherapy, meta-analysis

## Abstract

**Background:**

High recurrence rate after curative treatment is the major problem for hepatocellular carcinoma (HCC). Cytokine-induced killer cells (CIKs) therapy was extensively studied among HCC patients. However, the value of CIKs therapy was controversial. A meta-analysis was performed to investigate the efficacy of adjuvant CIKs after invasive treatments among HCC patients.

**Methods:**

We searched online for literatures studying sequential CIKs therapy for HCC patients. Recurrence-free survival (RFS), progress-free survival (PFS) and overall survival (OS) were set as the main endpoints. Both overall and subgroup analysis were accomplished.

**Results:**

A total of 12 clinical trials with 1,387 patients were included. The pooled analysis showed a significant improvement of RFS, PFS and OS in CIK group (HR 0.56, 95% CI 0.47-0.67, *p*<0.00001 for RFS; HR 0.53, 95% CI 0.40-0.69, *p*<0.00001 for PFS; HR 0.59, 95% CI 0.46-0.77, *p*<0.0001 for OS). The proportion of CD4^+^ T cells increased significantly, while CD8^+^ T cells decreased significantly after CIKs therapy (WMD 4.07, 95% CI 2.58-5.56, *p*<0.00001; WMD -2.84, 95% CI -4.67 to -1.01, *p*=0.002, respectively). No significant differences of adverse events between CIK and non-CIK group existed.

**Conclusions:**

Conventionally invasive therapies combined with CIKs therapy could improve the prognosis of HCC patients, especially for RFS and PFS, with mild side effects. Optimizing patient selection shall be the direction in future studies.

## INTRODUCTION

Disease recurrence after curative treatments is the major problem for hepatocellular carcinoma (HCC) [1]. The latent causes include failure to detect occult metastasis, tumor promoting microenvironment and lack of effective adjuvant treatments [2, 3]. To minimize disease recurrence, lots of strategies are employed including combination of local treatments, usage of antiviral agents and advanced imaging technology [4–6]. However, few consensus is achieved in the field of adjuvant systematic treatments including sorafenib, chemotherapy and immunotherapy [7].

Cytokine-induced killer cells (CIKs) consisting of activated NKG2D^high^ T cells, activated NK cells and NK T cells [8], were designed to alter suppressive immune microenvironment of tumor and consequently present certain efficacy in several malignancies [9–12]. Adoptive cells therapies (ACT) with CIKs were extensively studied among HCC patients in the Asia-Pacific region, where hepatitis B virus (HBV) related HCC constitute the major population of HCC. However, efficacy of this treatment on HCC patients are still controversial.

Therefore, we performed an update meta-analysis of clinical trials studying the efficacy of sequential CIKs treatments compared with non-CIK treatments in HCC, to provide comprehensive evidence of adjuvant efficacy of CIKs.

## RESULTS

### Literature research

**Figure 1:**
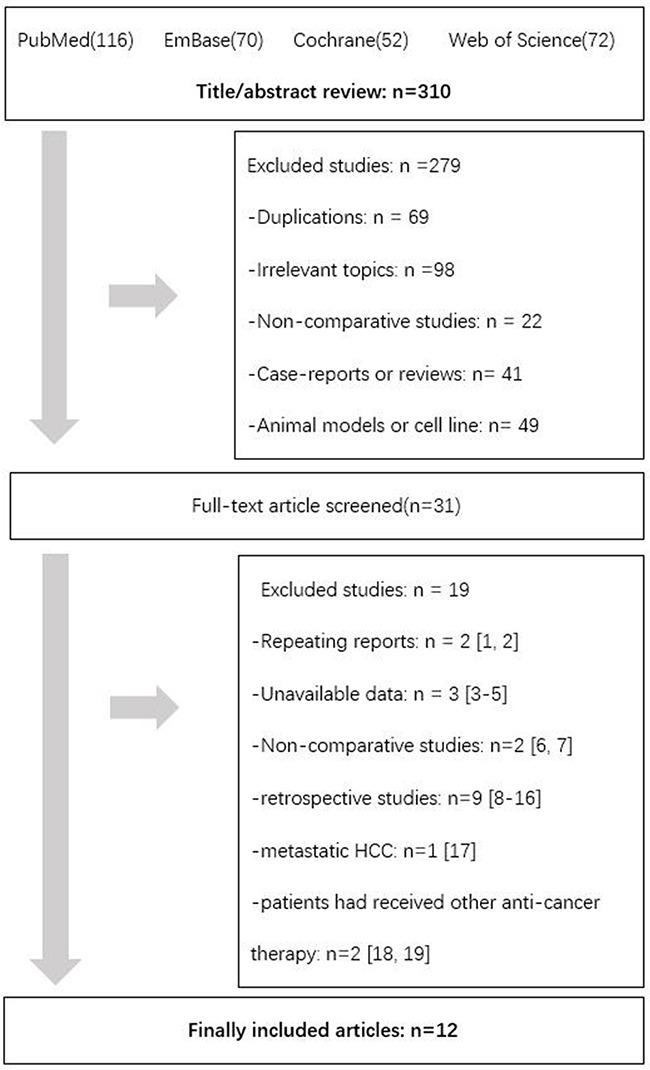
Flow diagram showing record identification, screening and study inclusion process

A total of 12 studies were included (Figure 1). After title and abstract review, 279 studies were excluded. Then comprehensively review excluded 19 studies (Appendix-Search Strategies and Excluded Studies). Screening of the references listed in related articles yielded no more studies available. We finally included 12 articles meeting the selection criteria into this meta-analysis involving 1,387 patients. Agreement between the two reviewers was 100% for study selection and 95.2% (4/84 was controversial) for quality assessment of the trials.

### Characteristics of eligible studies

Over all, we included 9 RCTs and 3 quasi-RCTs (Table 1). All of the eligible studies were conducted in Asia. Two studies were conducted in South of Korea [13] and Japan [14], and the rest of the included studies were conducted in China. Previous treatments included liver resection, TACE, RFA, PMCT and PEI. Yu et al [15] divided the patients into 3 groups: resection plus CIKs versus resection along, TACE plus CIKs versus TACE along, supportive care with CIKs versus supportive care along, all of which were labeled as Yu 2014-A, B and C respectively. Hui et al [16] compared resection plus 3 and 6 cycles of CIKs therapy versus resection along, which were labeled as Hui 2009-A for 3-cycles CIK group and Hui 2009-B for 6-cycles CIK group. Lee et al [13] conducted subgroup analysis between patients underwent different previous treatments, which were labeled as Lee 2015-A for resection group and Lee 2015-B for RFA/PEI group respectively. Eight studies used CIKs-only immunotherapy to treat patients in the study group. Other four studies evaluated the efficacy of CIK-based cytotherapy as follows: Xu et al [17] used dendritic cells (DCs), γδ T cells plus CIKs; Zhang et al [18] used DCs plus CIKs; Cui et al [19] used NK cells, CIKs and γδ T-cells. Qiu et al [20] used CIKs plus α-Gal epitope-expressing tumor cell-pulsed DCs. Infusions of CIKs were mostly via peripheral veins, while local tumor injections were also used in some studies. Various clinical stage systems were used: seven studies [14, 16–18, 20–22] used TNM stage system, while 3 studies [15, 19, 23] used BCLC stage system and the other two studies [13, 24] used American Joint Committee on Cancer Staging System (6th edition). Only three studies reported changes of lymphocyte subsets, two [15, 18] of which used peripheral venous blood and one [14] study used PBMCs. Overall, with a mean age of 46.1 years, most of the patients in the studies had a good performance status and more than 3-months life expectancy. With the exact number of male and female patients, 10 studies were summarized to a 6:1 male/female ratio (969/160). Baseline characteristics of the included patients in this meta-analysis showed no significant differences between CIK and non-CIK group (Supplementary Table 1).

**Table 1:** Baseline characteristics of the eligible studies

Study	Study period	Country	Study design	No. of patients (male/female)	Median follow-up	Clinical stage	Previous treatment	CIKs per cycle	Method of infusion
Takamaya 2000	1992-1995	Japan	RCT	150(NA)	4.4 years	TNM I/II/IIIA/IVA	resection	7.1×10^10^	5 infusion IV
Weng 2008	2002-2004	CHN	RCT	85(60/25)	NA	TNM I/II/IIIIA	TACE+RFA	1.0-1.5×10^10^	8-10 infusions via hepatic arteries
Hui 2009	2000-2002	CHN	RCT	127(97/30)	5-7 years	NA	resection	1-2×10^10^	3 or 6 infusions (1 per 2 weeks) IV
Hao 2010	2005-2008	CHN	quasi-RCT	146(129/17)	NA	BCLC A/B/C	TACE	1-5×10^10^	1-3 infusions (4 per 1 month) IV
Qiu 2011	NA	CHN	RCT	18(15/3)	16.8 months	TNM III	Surgery +radio/chemo-therapy	0.2-2×10^10^	2-7 infusions (1 per week)
†Wang 2012	2004-2006	CHN	quasi-RCT	76(66/10)	44(10-88) months	TNM I/II	TACE+RFA	1.0-1.5×10^10^	6-12 infusions (1 per 2 weeks) IV or via hepatic arteries
†Xu 2013	2008-2011	CHN	RCT	80(65/15)	6-36 months	TNM III	TACE+PMCT	DCs=1-1.2×10^8^ CIKs=γδ T cells=0.3-1.0×10^10^	2 cycles,1 cycle per month IV and local tumor injection
Yu 2014	2004-2009	CHN	RCT	132(116/16)	18.6 months	BCLC A/B/C	resection /TACE /Support	1.01×10^10^ (0.72-1.21 ×10^10^)	2–36 cycles (1 per 1 month)
†Zhang 2014	2008-2012	CHN	RCT	85(NA)	NA	TNM I/II	TACE+RFA	DCs=CIKs =1.0×10^10^	6 courses IV and local tumor injection
Cui 2014	2010-2011	CHN	quasi-RCT	62(47/15)	12 months	BCLC A/B/C	RFA	1.2-2.0×10^9^ (NK, CIK and γδ T cell)	3 or 6 courses (8 infusions per course) IV
Lee 2015	2008-2012	KOR	RCT	226(186/40)	36.5-40 months	I/II‡	resection/RFA /PEI	(6.4±2.1)×10^9^	16 infusions IV
Xu 2016	2008-2013	CHN	RCT	200(100/100)	38.2(3.7-73) months	T1/T2/T3a⊕	resection	1.0-1.5×10^10^	4 cycles IV

Abbreviations: 5-FU:5-fluoro-2,4(1h, 3h) pyrimidinedione; ADM: Doxorubicin Hydrochloride; CHN: country of China; CIK: cytokine induced killer (cell); DC: dendritic cell; DDP: cisplatin; EPI: epirubicin; FUDR: floxuridine; GEM: gemcitabine; HCPT: hydroxycamptothecin; IC: intracutaneous injection; IV: intravenous injection; KOR: country of Korea; MMC: mitomycin; NA: not available; NS: normal saline; OXA: oxaliplatin; PEI: percutaneous ethanol injection; PMCT: percutaneous microwave coagulation therapy; quasi-RCT: quasi-randomized controlled trial; RCT: randomized controlled trial; RFA: radiofrequency ablation; TACE: transcatheter arterial chemoembolization; THP: pirarubicin; UFL: ultra fluid lipiodol.

†articles published in Chinese; ‡: Stage based on American Joint Committee on Cancer staging system (6th edition); ⊕: Stage based on American Joint Committee on Cancer staging system (7th edition).

### Methodological assessment of included articles

Quality of the eligible studies was summarized (Supplementary Table 2 for detailed assessment of individual studies; Supplementary Figure 1 and 2 for risk of bias in individual studies and among the included studies, respectively). The included quasi-RCTs assigned the allocation depending on patients’ choices. But for RCTs, adequate sequence generation was well done in seven studies [13–17, 20, 24], while not mentioned clearly in others [18, 19, 21–23]. Only three studies [13, 15, 24] provided details of allocation concealment and two [13, 19] showed prognostic imbalance between CIK and non-CIK groups. All the included studies were free of selective reporting. Taken together, five studies [14, 16, 17, 20, 24] without any bias of high-risk were judged as high quality.

### Primary outcomes

**Figure 2:**
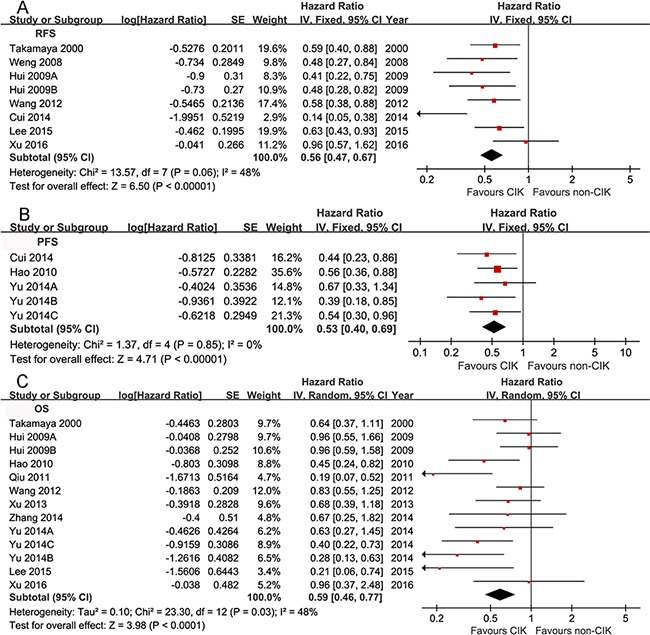
Comparison of RFS (A), PFS (B) and OS (C) between CIK and non-CIK groups The fixed effects meta-analysis model (Mantel-Haenszel method) was used for RFS **A**. and PFS **B**. while the random-effects model (Mantel-Haenszel method) was used for OS **C**.. Each trial is represented by a square, the center of which gives the HR for that trial. The size of the square is proportional to the information provided by the trial report. The ends of the horizontal bars denote the 95% CI.

The pooled analysis showed a significant improvement of RFS, PFS and OS in CIK group (HR 0.56, 95% CI 0.47-0.67, *p*<0.00001 for RFS; HR 0.53, 95% CI 0.40-0.69, *p*<0.00001 for PFS; HR 0.59, 95% CI 0.46-0.77, *p*<0.0001 for OS) (Figure 2).

The analysis showed a significant benefit of RFS in CIK group, indicating a 44% reduction in the relative risk of recurrence with no statistical heterogeneity (*p* = 0.06, *I^2^* = 48%). However, in subgroup analysis of study designs, no significant amelioration of RFS was found in CIK group but a greater heterogeneity was observed among quasi-RCTs (HR 0.30, 95% CI 0.07-1.24, *p* = 0.1; *p* = 0.01, *I^2^* = 85% for heterogeneity). In other subgroups, such as study quality, tumor staging systems, clinical characteristics, previous treatments and CIKs treatments, the analysis showed a consistency of RFS with that in overall analysis.

Only three studies [15, 19, 23] reported PFS and were analyzed to show a statistically significant delaying of disease progression without remarkable heterogeneity between these studies (*p* = 0.85, *I^2^* = 0). Subgroup analysis also showed a consistency of PFS with that in overall analysis.

OS was significantly improved in CIK group. However, the heterogeneity among the included studies was statistically significant (*p* = 0.03, *I^2^* = 48). Nevertheless, the heterogeneity of OS was not significant in subgroup analysis of study quality, tumor staging systems, and clinical characteristics of the enrolled patients, all of which may account for the heterogeneity in the overall analysis. Moreover, significant improvement of OS in CIK group was observed in all of the above subgroups. But in subgroup of resection as the previous treatments, more than 5 times injection of CIKs as the cytotherapy, and AJCC as the staging system, there was no clear evidence of prolongation of OS in CIK group (The outcomes of both overall and subgroup analysis were summarized in Supplementary Table 3 and 4, respectively).

### Secondary outcomes

#### Changes of lymphocyte subsets

**Figure 3:**
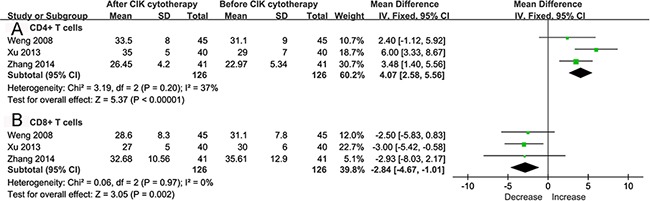
Forest plot for lymphocyte subsets assessment The outcomes were obtained from patients before and after CIK cytotherapy. The fixed effects meta-analysis model (Mantel-Haenszel method) was used for CD4^+^ T cells **A**. and CD8^+^ T cells **B**. in this analysis.

Only 3 studies [17, 18, 21] provided available data about alteration of lymphocyte subsets. The analysis demonstrated that the proportion of CD4^+^ T cells increased significantly after CIKs therapy, while CD8^+^ T cells decreased significantly (WMD 4.07, 95% CI 2.58-5.56, *p*<0.00001; WMD -2.84, 95% CI -4.67 to -1.01, *p* = 0.002, respectively). The heterogeneity among these 3 studies was not statistically significant (*p* = 0.20, *I^2^* = 37% for CD4^+^ T cells; *p* = 0.97, *I^2^* = 0 for CD8^+^ T cells, respectively) (Figure 3).

#### Adverse events

**Figure 4:**
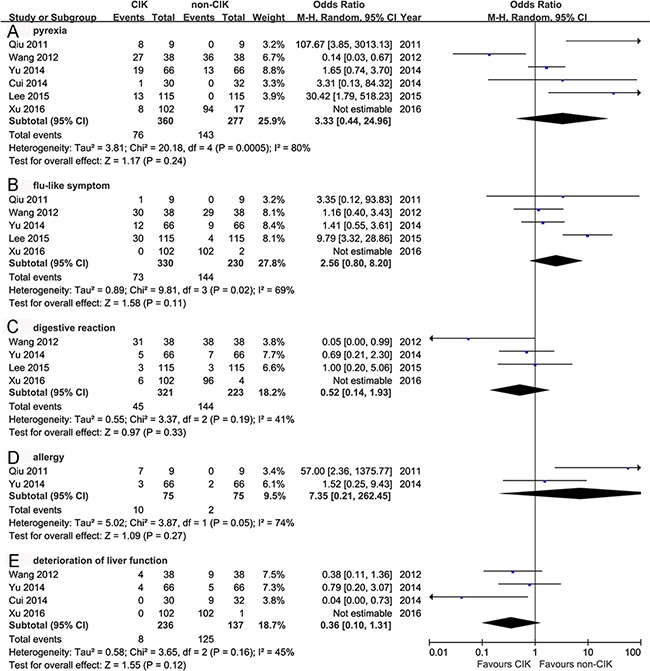
Comparison of the adverse events between CIK group and non-CIK group The random-effects meta-analysis model (Mantel-Haenszel method) was used in this analysis.

The pooled analysis exhibited no significant differences of AEs between CIK and non-CIK groups (Figure 4). Most studies reported no severe AEs in CIK group but without quantitative details. Lee et al [13] reported that AEs in Grade 3/4 were 6% (7/115) and 4% (4/115) in CIK and non-CIK groups, respectively (*p* = 0.354). While 0% (0/66) and 5% (3/66) were reported by Yu et al [15] and none of the AEs was associated with CIK cytotherapy. Pyrexia especially slight fever, was mostly reported. Other AEs such as flu-like symptom, digestive adverse reaction, allergy, and deterioration of liver function, showed no significant differences between these two groups.

### Sensitivity analysis

Sensitivity analysis suggested that no individual studies dominantly affected the pooled HRs for RFS, PFS and OS, indicating that the results of this meta-analysis were statistically stable (Supplementary Figure 3).

### Publication bias

No significant publication bias for RFS, PFS, OS, changes of lymphocyte subsets and AEs was observed in Begg's funnel plot (Supplementary Figure 4).

## DISCUSSION

Reversing the immune-suppressive microenvironment in tumor shall be a promising strategy for HCC [25]. ACT as a potentially effective treatment, contains several types of lymphocytes including tumor-infiltrating lymphocytes, lymphokine-activated killer cells, and CIKs, etc. First reported by Schimidt Wolf and his colleagues, CIKs were tested in various cancers, which remained controversial for the heterogeneity of individual immnuotolerance mechanism and lack of consensus on the selection of right patients at right time [26]. In the present meta-analysis, CIKs were indicated as an effective adjuvant therapy for HCC patients by improving RFS and PFS.

We found that CIKs prolonged RFS and PFS with reasonable heterogeneity between studies. In subgroups of quasi-RCTs, only two studies reported RFS, which may result in insignificant RFS as well as heterogeneity between-studies for the limited number of available studies. In other subgroups, such as study quality, tumor staging systems, clinical characteristics, previous treatments and CIKs treatments, the analysis showed consistency of both RFS and PFS. Liver resection and other invasive treatments massively reduced tumor burden and alleviate pre-existing immune suppression, which might improve the effect of CIKs. Moreover, transarterial embolization or local ablation may increase tumor immunogenicity and unmask tumor-specific antigens [27, 28]. After curative resection or other local therapies, HCC patients without metastases or portal venous thrombus would benefit from sequential CIKs transplantation in terms of cancer recurrence and progression.

The significant improvement of OS by CIKs was not confirmed due to the defects of heterogeneity between studies. After subgroup analysis, we found that the heterogeneity may result from differences in study quality, tumor staging systems, and clinical characteristics of the enrolled patients. And significant improvement of OS by CIKs was observed in all of the above subgroups. But in subgroup of resection as the previous treatments, more than 5 times injection of CIKs as the cytotherapy, and AJCC as the staging system, there was no significant prolongation of OS in CIK group. For only two studies using AJCC staging system, more convincing evidence should be collected in further clinical studies. So far, HCC patients with sufficient liver function (Child-Pugh A/B) and without metastases or portal venous thrombus, would have a further prolonged OS by CIKs after local therapies (e.g. TACE/RFA/PEI/PMCT). However, these patients would not have a further improvement of OS by CIKs after liver resection. According to our previous studies, the OS of HCC patients was significantly influenced by treatments of later lines, which made it difficult to improve OS for CIKs [29]. Thus, RFS and PFS shall be more appropriate endpoints for evaluating the efficacy of CIKs. Summarily, sequential CIKs presented a promising efficacy in improving RFS and PFS for HCC patients after receiving conventional procedures, and in improving OS for those underwent TACE/RFA/PEI/PMCT.

Monitoring the effect of CIKs during the treatment was also essential. Several studies investigated the predictive value of peripheral lymphocyte subsets during CIKs treatment, of which the results were inconsistent [30]. It's reported that CD4^+^ T cells were required for the remodeling of the tumor microenvironment to sustain tumor regression [31]. Moreover, Endig, et al found depletion of CD8^+^ T cells markedly delays tumor progression in mice with chronic liver injury, which indicated a facilitative role of CD8^+^ T cells in the tumor microenvironment of HCC [32]. In present study, the proportion of CD4^+^ T cells increased significantly while CD8^+^ T cells decreased dramatically in patients receiving CIKs therapy, which implied potential predictors for CIKs. But further clinical confirmation was needed due to a limited number of trials reported changes of lymphocyte subsets in this study.

CIK cytotherapy presented a reliable safety profile without increase of AEs. Many studies didn't provide completely quantitative information of AEs, but they did report that AEs in Grade 3/4 were rare. Based on our results, we deduced that CIKs might be more salutary for HCC patients with sufficient liver function (Child-Pugh A/B) and without metastasis or portal venous thrombus. However, the optimal timing of sequential CIKs cannot be concluded because a wide range of treatment cycles were conducted among the included studies. Randomized clinical trials were needed to further confirm the timing of CIKs treatments.

The present study employed a more advanced and accurate statistical strategy to achieve more conclusive results. Antecedent meta-analysis assessed the efficacy of CIKs for HCC patients after minimally invasive treatments and concluded that both RFS and OS were significantly improved in CIKs group compared with control group [33]. They included both prospective and retrospective studies, and pooled survival rate with OR to evaluate the efficacy of CIK cytotherapy. By comparison, our study possessed some advantages. Firstly, we included only prospective studies (RCTs and quasi-RCT), and performed a stratification analysis. Though the number of potentially eligible studies was limited, we evaluated strictly on the quality of the included studies by well-accepted tools. Secondly, to integrate time-to-events (RFS, PFS and OS), we calculated HR to show a trend over time rather than OR to show efficacy at some time point. Thirdly, both survival outcomes, changes of lymphocyte subsets and AEs were analyzed.

Our study also had some limitations. Primarily, all eligible clinical trials were conducted in oriental countries, where chronic HBV infection was the major etiology. Thus, our results could not be easily expanded to HCV-related HCC and other types of HCC. Secondly, a limited number of studies were included in secondary outcomes. Though these studies reported relatively consistent results in secondary outcomes, more eligible studies were needed to draw a more convincing conclusion. Lastly, the present meta-analysis was not based on individual patient data and unable to subject to an open external evaluation procedure. Therefore, the analysis may have potential bias in over-estimating the treatment effects. Several completed clinical trials haven't display their results at present, which limited our collection of concerning data (NCT01749865). Despite these limitations, CIKs as an adjuvant therapy for HCC presented a potentially efficacy, which deserved further verification in more specific subgroup of HCC patients.

Above all, sequential CIKs presented a promising efficacy in improving RFS and PFS for HCC patients after receiving conventionally invasive therapies, and in improving OS for those after TACE/RFA/PEI/PMCT. Optimizing patient selection shall be the direction to promote the efficacy of CIKs in future studies.

## MATERIALS AND METHODS

### Study protocol and literature search

A prospective protocol of objectives, search strategies, selection criteria, outcome measurements, and methods of statistical analysis were settled down in advance, which was accordant with Preferred Reporting Items for Systematic Reviews and Meta-Analyses guidelines and conducted in accordance with the Cochrane Collaboration's systematic review framework [34, 35].

With no language or regional restrictions, we searched on Pubmed, Embase, Web of Science and the Cochrane Library using key words as hepatocellular carcinoma and cytokine-induced killer cells date to October 7, 2016. The trial register and reference lists of related articles were also searched for supplements. We regularly updated the search until October 7, 2016. We tried to obtain information about relevant studies that have been completed but never published in the following ways: 1) The International Standard Randomized Controlled Trial Number Register scheme; 2) The International Clinical Trials Registry Platform Search Portal; 3) Formal letters of request for information about unpublished studies to colleagues.

### Selection criteria

Inclusion criteria: 1) patients diagnosed confirmedly of HCC without systematic treatments before enrolling; 2) studies comparing the outcomes of conventional treatments plus sequential CIKs with conventional treatments alone; 3) conventional treatments including liver resection, TACE, percutaneous microwave coagulation therapy (PMCT), radiofrequency ablation (RFA), percutaneous ethanol injection (PEI); and 4) both randomized controlled trials (RCTs) and quasi-RCTs with available data were included.

Exclusion criteria: 1) patients with metastatic HCC or mixed malignancies; 2) repeated reports, which were excluded in several ways: same studies published in different magazines; overlapped data from the studies reported by the same authors or from the same organization.

### Data extraction

Assessment of the eligibility of the retrieved studies and data extraction of the included studies were performed by Xiu-Rong Cai and Xing Li separately. A unified data form was applied, which included the following items: first author, publication year, study period and country, study design, patient demographic, disease characteristics, details of treatments, hazard ratio (HR) with 95% confidential interval (95% CI) for the CIK group compared with non-CIK group, changes of lymphocyte subsets before and after CIKs therapy and adverse events (AEs) of both groups. Any discrepancies were discussed mutually and ultimately solved by Xiu-Rong Cai. The same method was used to evaluate the quality of the included studies.

### Outcome definition

Our primary outcomes were recurrence-free survival (RFS), progress-free survival (PFS) and overall survival (OS). Secondary outcomes were changes of lymphocyte subsets and AEs. RFS was defined as the time from treatments to either local, distant recurrence or death (any cause). PFS was defined as the time from treatments to the date on disease progressing or death (any cause). With OS defined as the time from treatments to death or to the date of the last follow-up for censored patients. Changes of lymphocyte subsets such as CD4^+^ T cells and CD8^+^ T cells were recorded before and after CIKs. AEs were classified and graded according to the Common Terminology Criteria for Adverse Events, version 3.0.

### Qualitative assessment

The methodological quality of RCTs and quasi-RCTs were assessed by the Cochrane risk of bias tool [36], which contained bias of sequence generation, allocation concealment, blinding, incomplete outcome data addressed, selective reporting and other bias. Each bias was labeled as low-risk, unclear-risk or high-risk. A study without any high-risk of bias was judged as high-quality RCT.

### Statistical analysis

We proceeded all meta-analysis based on Review Manager version 5.0 and STATA SE version 12.0.

RFS, PFS and OS were pooled by HR with 95% CI, which was estimated by the tool introduced by Parmar MK and his colleagues [37]. HR of less than 1 represented a benefit of CIK group compared with non-CIK group. Continuous and dichotomous variables were denoted by weighted mean difference (WMD) and odds ratio (OR) with 95% CI. OR greater than 1 showed more frequency of events in CIK group. All reported *p* values were two-tailed and deemed statistically significant if less than 0.05.

Both *χ^2^* test and *I^2^* tests were utilized to evaluate heterogeneity among studies. When the *p* value from the heterogeneity analysis was greater than 0.1, a fixed effects model was applied, which demonstrated no significant heterogeneity. Otherwise a random-effects model was applied [38]. Specifically, the higher *χ^2^* and *I^2^* statistic, the greater heterogeneity.

Subgroup analysis was performed considering different study designs, study quality, tumor staging systems, clinical characteristics, previous treatments and details of CIK treatments. To assess the consistency of the results and evaluate the influence of single studies on overall risk estimate, we conducted a sensitivity analysis by omitting each study in turn. Begg's funnel plot was used to analyze publication bias. The *p* value less than 0.05 represented a statistically significant publication bias.

## SUPPLEMENTARY MATERIALS FIGURES AND TABLES














