# Supplementary material for: Autologous transplantation of cytokine-induced killer cells as an adjuvant therapy for hepatocellular carcinoma in Asia: an update meta-analysis and systematic review
Source: Oncotarget. 2017 Feb 17;8(19):31318–28. doi: 10.18632/oncotarget.15454 (PMC5458210; doi:10.18632/oncotarget.15454)
Supplement: Supplementary file 1 [file oncotarget-08-31318-s001.pdf]

Autologous transplantation of cytokine-induced killer cells as an adjuvant therapy for hepatocellular carcinoma in Asia: an update meta-analysis and systematic review

SUPPLEMENTARY FIGURES AND TABLES

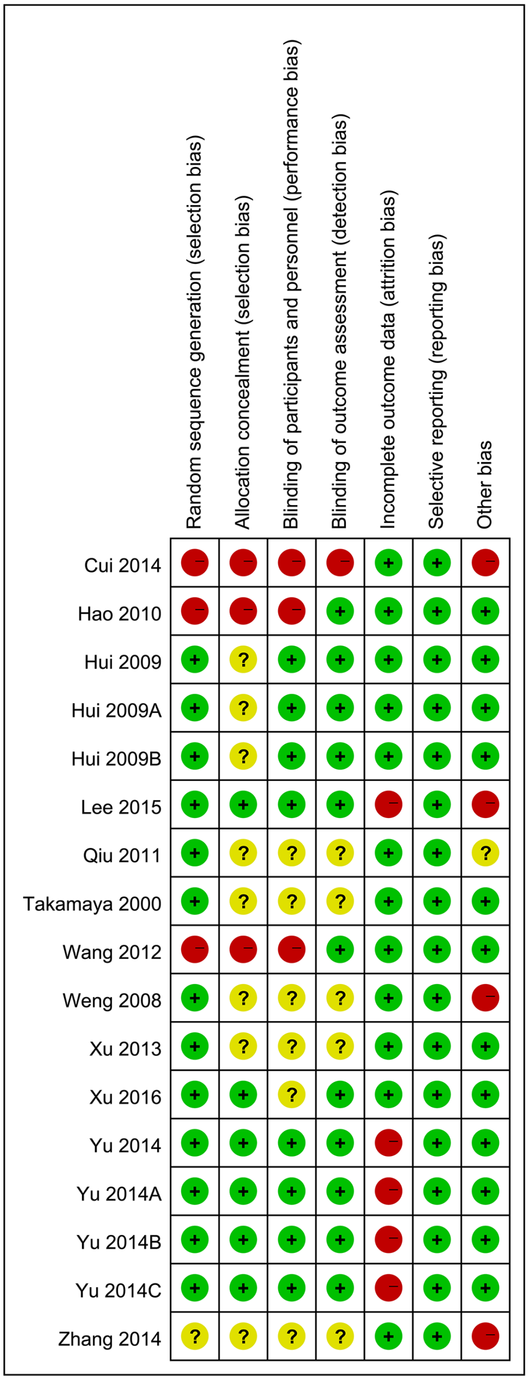

Supplementary Figure 1: Risk of bias in individual studies assessed by review authors using the Cochrane risk of bias tool. Each color represented a different level of bias: red for high-risk, green for low-risk, and yellow for unclear-risk of bias, respectively.

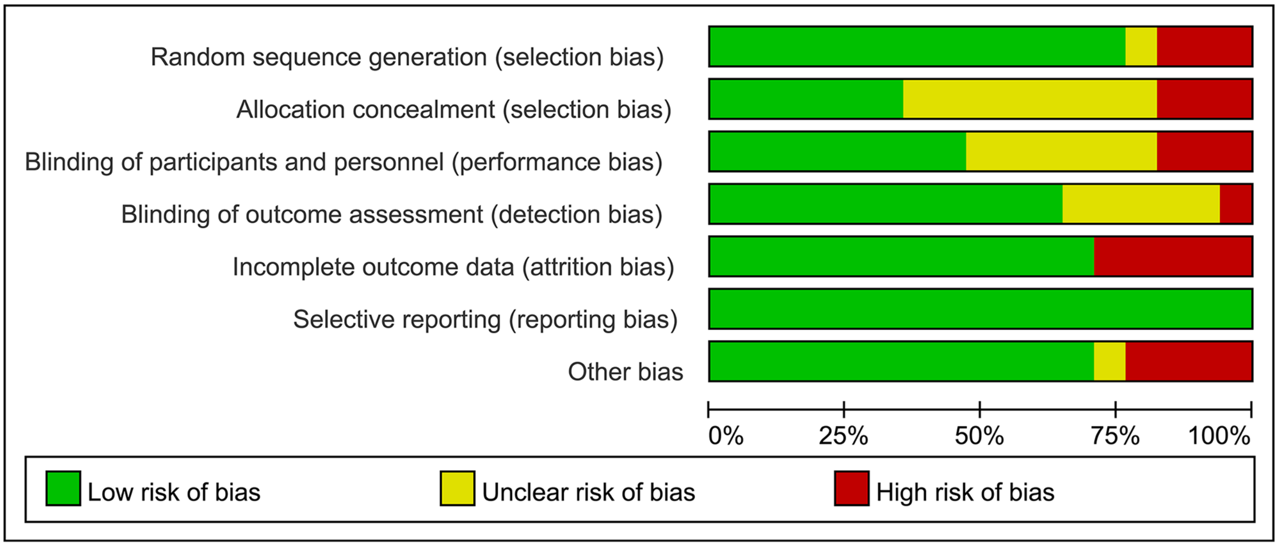

Supplementary Figure 2: Summary for risk of bias among the included studies. Each risk of bias item was presented as percentages across all included studies. Different colors represented a different level of bias: red for high-risk, green for low-risk, and yellow for unclear-risk of bias, respectively.

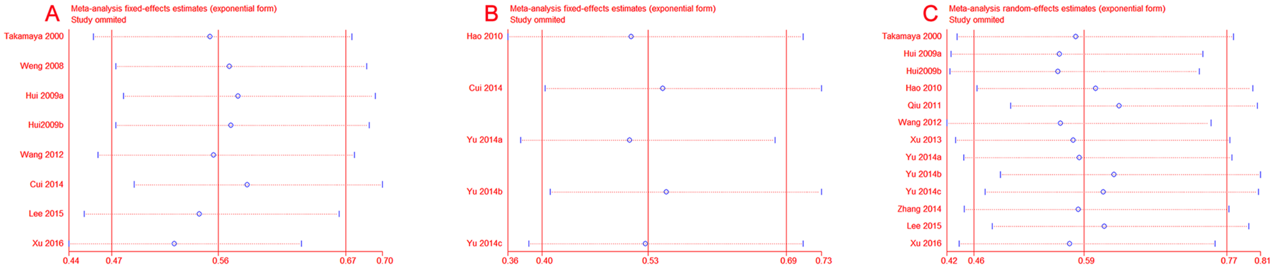

Supplementary Figure 3: Sensitivity analysis for the effect of each individual study on the pooled HRs. HRs with 95% CI were calculated from the rest of the studies after eliminating one study, and were presented respectively as the circle combining the dotted line in each row. The eliminating study was listed on the Y-axis, and the pooled HR with 95% CI were marked on the X-axis. The fixed effects meta-analysis model was used for RFS **A.** and PFS **B.** while the random-effects model was used for OS **C.**.

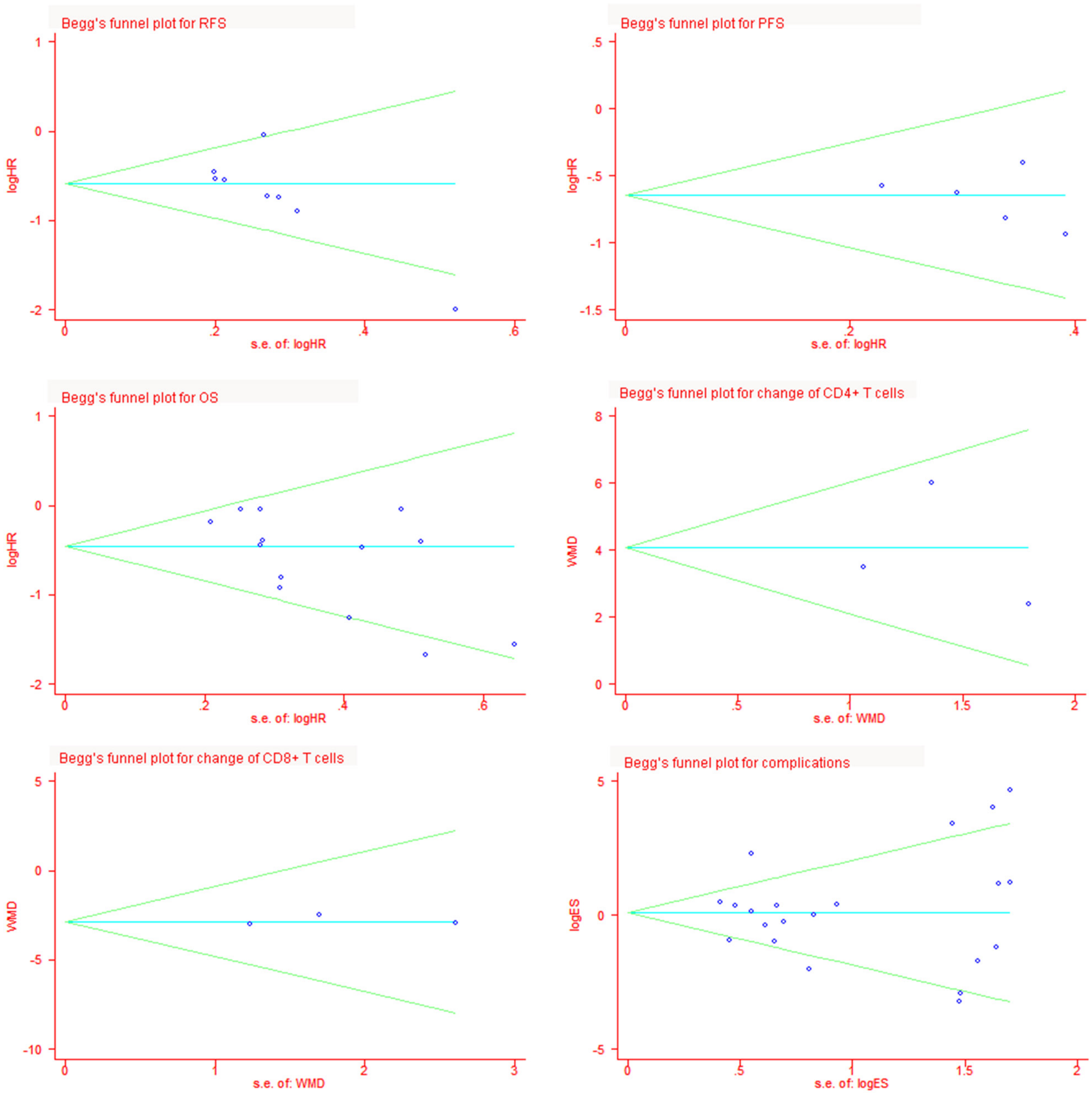

Supplementary Figure 4: Publication bias of different outcomes: RFS, PFS, OS, changes in lymphocyte subsets, and adverse events. Begg's funnel plots were used to assess the potential publication bias. A two-tailed *p* value under 0.05 was deemed statistically significant.

Supplementary Table 1: Baseline characteristics of the included patients in the meta-analysis.

See Supplementary File 1

Supplementary Table 2: The Cochrane collaboration's tool for assessing risk of bias.

See Supplementary File 2

Supplementary Table 3: Summary for the outcomes of patients in CIK group compared with non-CIK group.

See Supplementary File 3

Supplementary Table 4: Summary for the subgroup analysis.

See Supplementary File 4

Appendix 1: Appendix-Excluded Studies.

See Appendix 1

Appendix 2: Appendix-Search Strategies.

See Appendix 2
