# Supplementary material for: Autologous transplantation of cytokine-induced killer cells as an adjuvant therapy for hepatocellular carcinoma in Asia: an update meta-analysis and systematic review
Source: Oncotarget. 2017 Feb 17;8(19):31318–28. doi: 10.18632/oncotarget.15454 (PMC5458210; doi:10.18632/oncotarget.15454)
Supplement: Supplementary file 2 [file oncotarget-08-31318-s002.docx]

**Supplemental Table 1. Baseline characteristics of the included patients in the meta-analysis.**

|  |  | CIK | | non-CIK | |
| --- | --- | --- | --- | --- | --- |
| Subgroup | | n | % | n | % |
| Hepatitis | HBV | 462 | 64.6 | 432 | 60.4 |
|  | HCV | 62 | 8.7 | 62 | 8.7 |
|  | HBV+HCV | 2 | 0.3 | 2 | 0.3 |
|  | other hepatitis | 90 | 12.6 | 94 | 13.1 |
|  | unknown | 99 | 13.8 | 82 | 11.5 |
| Cirrhosis | Yes | 314 | 43.9 | 279 | 39.0 |
|  | No | 164 | 22.9 | 154 | 21.5 |
|  | unknown | 237 | 33.1 | 239 | 33.4 |
| Liver function  (Child–Pugh Score) | A | 579 | 81.0 | 529 | 74.0 |
|  | B | 93 | 13.0 | 99 | 13.8 |
|  | unknown | 43 | 6.0 | 44 | 6.2 |
| Tumor size, cm | ≤3 | 322 | 45.0 | 303 | 42.4 |
|  | 3-5 | 189 | 26.4 | 167 | 23.4 |
|  | ≥5 | 185 | 25.9 | 175 | 24.5 |
|  | unknown | 19 | 2.7 | 27 | 3.8 |
| Clinical stage | BCLC Stage A | 25 | 3.5 | 29 | 4.1 |
|  | BCLC Stage B | 53 | 7.4 | 29 | 4.1 |
|  | BCLC Stage C | 90 | 12.6 | 95 | 13.3 |
|  | AJCC Stage I | 98 | 13.7 | 94 | 13.1 |
|  | AJCC Stage Ⅱ | 16 | 2.2 | 18 | 2.5 |
|  | AJCC T1 | 92 | 12.9 | 93 | 13.0 |
|  | AJCC T2 | 4 | 0.6 | 3 | 0.4 |
|  | AJCC T3a | 4 | 0.6 | 4 | 0.6 |
|  | TNM Stage Ⅰ/Ⅱ | 81 | 11.3 | 85 | 11.9 |
|  | TNM StageⅢ/Ⅳ | 76 | 10.6 | 73 | 10.2 |
|  | unknown | 176 | 24.6 | 149 | 20.8 |
| Total | 1387 | 715 | 100.0 | 672 | 100.0 |
