# Supplementary material for: Autologous transplantation of cytokine-induced killer cells as an adjuvant therapy for hepatocellular carcinoma in Asia: an update meta-analysis and systematic review
Source: Oncotarget. 2017 Feb 17;8(19):31318–28. doi: 10.18632/oncotarget.15454 (PMC5458210; doi:10.18632/oncotarget.15454)
Supplement: Supplementary file 3 [file oncotarget-08-31318-s003.docx]

**Supplemental Table 2. The Cochrane collaboration’s tool for assessing risk of bias.**

| study | adequate sequence generation | allocation concealment | blinding | incomplete outcome data addressed | free of selective reporting | free of other bias | description of other bias |
| --- | --- | --- | --- | --- | --- | --- | --- |
| Takamaya 2000 | Yes | Unclear | No | No | Yes | Yes | Use of intention-to-treatment analysis |
| Weng 2008 | Unclear | Unclear | Unclear | No | Yes | Yes | poorly described the design of study |
| Hui 2009 | Yes | Unclear | Yes | No | Yes | Yes | Use of intention-to-treatment analysis and single-blinded method without details. |
| Hao 2010 | No | No | No | No | Yes | No | Use of intention-to-treatment analysis and insufficient details. |
| Qiu 2011 | Yes | Unclear | Unclear | No | Yes | No | poorly described the design of study |
| Wang 2012 | No | No | No | No | Yes | No | Patient's decision-based assignment and use of intention-to-treatment analysis |
| †Xu 2013 | Yes | Unclear | Unclear | No | Yes | Yes |  |
| Cui 2014 | No | No | Unclear | No | Yes | No | Patient's decision-based assignment; Prognosis imbalanced group (age in CIK group was higher than that in non-CIK group);1/30 of patients in the CIK group was lost to follow up. |
| Yu 2014 | Yes | Yes | Yes | No | Yes | Yes |  |
| Zhang 2014 | Unclear | Unclear | Unclear | No | Yes | No | poorly described the design of study |
| Joon Hyeok Lee 2015 | Yes | Yes | Yes | No | Yes | No | Use of intention-to-treatment analysis in efficacy analysis; Prognosis imbalanced group; Industry funding |
| Xu 2016 | Yes | Yes | Yes | No | Yes | No | Use of intention-to-treatment analysis in efficacy analysis; The investigators and the patients, but not the physicians, radiologists or statisticians were aware of the intervention assignments. |
