# Supplementary material for: Autologous transplantation of cytokine-induced killer cells as an adjuvant therapy for hepatocellular carcinoma in Asia: an update meta-analysis and systematic review
Source: Oncotarget. 2017 Feb 17;8(19):31318–28. doi: 10.18632/oncotarget.15454 (PMC5458210; doi:10.18632/oncotarget.15454)
Supplement: Supplementary file 4 [file oncotarget-08-31318-s004.docx]

**Supplementary Table 3. Summary for the outcomes of patients in CIK group compared with non-CIK group.**

| variables | No. of patients | | | | HR/WMD/OR(95%CI) | heterogeneity of studies | | | | | |
| --- | --- | --- | --- | --- | --- | --- | --- | --- | --- | --- | --- |
|  | **No. of studies** | **CIK** | **non-CIK** | **total** |  | ***P* value** | ***X^2^*** | **df** | ***I²*(%)** | ***P* value** | **analysis model** |
| Survival data | | | | | | | | | | | |
| RFS | 7 | 487 | 439 | 926 | 0.56 [0.47, 0.67] | 0.00001^****^ | 13.57 | 7 | 48 | 0.06 | Fixed |
| PFS | 3 | 168 | 172 | 340 | 0.53 [0.40, 0.69] | 0.00001^****^ | 1.37 | 4 | 0 | 0.85 | Fixed |
| OS | 10 | 640 | 600 | 1240 | 0.59 [0.46, 0.77] | 0.0001^***^ | 23.3 | 12 | 48 | 0.03 | Random |
| Immunocyte subsets | | | | | | | | | | | |
| CD4+ T cells | 3 | 126 | 124 | 250 | 4.07 [2.58, 5.56] | 0.00001^****^ | 3.19 | 2 | 37 | 0.2 | Fixed |
| CD8+ T cells | 3 | 126 | 124 | 250 | -2.84 [-4.67, -1.01] | 0.002^*^ | 0.06 | 2 | 0 | 0.97 | Fixed |
| Complications | | | | | | | | | | | |
| pyrexia | 6 | 357 | 357 | 714 | 3.33 [0.44, 24.96] | 0.24 | 20.18 | 4 | 80 | 0.0005 | Random |
| flu-like symptom | 5 | 327 | 325 | 652 | 2.56 [0.80, 8.20] | 0.11 | 9.81 | 3 | 69 | 0.02 | Random |
| digestive reaction | 4 | 318 | 316 | 634 | 0.52 [0.14, 1.93] | 0.33 | 3.37 | 2 | 41 | 0.19 | Random |
| allergy | 2 | 75 | 75 | 150 | 7.35 [0.21, 262.45] | 0.27 | 3.87 | 1 | 74 | 0.05 | Random |
| deterioration of liver-function | 4 | 234 | 236 | 470 | 0.36 [0.10, 1.31] | 0.12 | 3.65 | 2 | 45 | 0.16 | Random |
