# Supplementary material for: Autologous transplantation of cytokine-induced killer cells as an adjuvant therapy for hepatocellular carcinoma in Asia: an update meta-analysis and systematic review
Source: Oncotarget. 2017 Feb 17;8(19):31318–28. doi: 10.18632/oncotarget.15454 (PMC5458210; doi:10.18632/oncotarget.15454)
Supplement: Supplementary file 5 [file oncotarget-08-31318-s005.docx]

**Supplementary Table 4. Summary for the subgroup analysis.**

| subgroup | variables | No. of studies | No. of patients | | | HR [95%CI] | *P* value | heterogeneity of studies | | | | |
| --- | --- | --- | --- | --- | --- | --- | --- | --- | --- | --- | --- | --- |
|  |  |  | **CIK** | **non-CIK** | **total** |  |  | ***X^2^*** | **df** | ***I²*(%)** | ***P* value** | **analysis model** |
| Study design | | | | | | | | | | | | |
| RCTs | RFS | 5 | 419 | 369 | 788 | 0.59 [0.48, 0.71] | 0.00001^****^ | 5.98 | 5 | 16 | 0.31 | Fixed |
|  | PFS | 2 | 96 | 98 | 194 | 0.51 [0.36, 0.71] | 0.0001^***^ | 1.24 | 3 | 0 | 0.74 | Fixed |
|  | OS | 7 | 489 | 444 | 933 | 0.56 [0.40, 0.79] | 0.0007^**^ | 20.3 | 9 | 56 | 0.02 | Random |
| quasi-RCTs | RFS | 2 | 68 | 70 | 138 | 0.30 [0.07, 1.24] | 0.1 | 6.6 | 1 | 85 | 0.01 | Random |
|  | OS | 3 | 151 | 156 | 307 | 0.68 [0.50, 0.94] | 0.02 | 2.72 | 2 | 27 | 0.26 | Fixed |
| Study quality | | | | | | | | | | | | |
| high-quality | RFS | 3 | 260 | 217 | 477 | 0.59 [0.46, 0.76] | 0.0001^***^ | 5.35 | 3 | 44 | 0.15 | Fixed |
|  | OS | 5 | 309 | 266 | 575 | 0.75 [0.58, 0.96] | 0.02 | 9.67 | 5 | 48 | 0.09 | Fixed |
| low-quality | RFS | 4 | 227 | 222 | 449 | 0.53 [0.41, 0.68] | 0.00001^****^ | 7.84 | 3 | 62 | 0.05 | Fixed |
|  | OS | 4 | 290 | 290 | 580 | 0.53 [0.41, 0.69] | 0.00001^****^ | 10.3 | 5 | 52 | 0.07 | Fixed |
| Tumor staging systems | | | | | | | | | | | | |
| TNM | RFS | 4 | 243 | 195 | 438 | 0.53 [0.42, 0.65] | 0.00001^****^ | 1.43 | 4 | 0 | 0.84 | Fixed |
|  | OS | 6 | 288 | 248 | 536 | 0.75 [0.61, 0.93] | 0.01 | 9.69 | 6 | 38 | 0.14 | Fixed |
| BCLC A/B/C | OS | 2 | 138 | 140 | 278 | 0.42 [0.30, 0.59] | 0.00001^****^ | 1.9 | 3 | 0 | 0.59 | Fixed |
| AJCC | RFS | 2 | 214 | 212 | 426 | 0.73 [0.54, 1.00] | 0.05 | 1.6 | 1 | 38 | 0.21 | Fixed |
|  | OS | 2 | 214 | 212 | 426 | 0.56 [0.26, 1.19] | 0.13 | 3.58 | 1 | 72 | 0.06 | Fixed |
| clinical characteristics | | | | | | | | | | | | |
| vascular invasion | RFS | 3 | 260 | 217 | 477 | 0.59 [0.46, 0.76] | 0.0001^***^ | 5.35 | 3 | 44 | 0.15 | Fixed |
|  | OS | 4 | 326 | 283 | 609 | 0.67 [0.53, 0.86] | 0.001 | 11.6 | 6 | 48 | 0.07 | Fixed |
| no metastasis or PVT | RFS | 5 | 328 | 278 | 606 | 0.51 [0.42, 0.63] | 0.00001^****^ | 8.69 | 5 | 42 | 0.12 | Fixed |
|  | OS | 5 | 387 | 347 | 734 | 0.70 [0.54, 0.91] | 0.007^**^ | 8.56 | 5 | 42 | 0.13 | Fixed |
| Liver function | | | | | | | | | | | | |
| Child-Pugh A | RFS | 2 | 214 | 212 | 426 | 0.73 [0.54, 1.00] | 0.05 | 1.6 | 1 | 38 | 0.21 | Fixed |
|  | OS | 2 | 214 | 212 | 426 | 0.56 [0.26, 1.19] | 0.13 | 3.58 | 1 | 72 | 0.06 | Fixed |
| Child-Pugh A/B | RFS | 5 | 273 | 227 | 500 | 0.50 [0.40, 0.61] | 0.00001^****^ | 7.85 | 5 | 36 | 0.16 | Fixed |
|  | OS | 6 | 376 | 335 | 711 | 0.67 [0.55, 0.81] | 0.0001^***^ | 13.8 | 8 | 42 | 0.09 | Fixed |
| Previous treatments | | | | | | | | | | | | |
| resection | RFS | 4 | 292 | 255 | 547 | 0.60 [0.47, 0.75] | 0.0001^***^ | 5.38 | 4 | 26 | 0.25 | Fixed |
|  | OS | 4 | 288 | 245 | 533 | 0.83 [0.63, 1.09] | 0.18 | 2 | 4 | 0 | 0.74 | Fixed |
| other local treatments | RFS | 4 | 195 | 184 | 379 | 0.51 [0.40, 0.67] | 0.00001^****^ | 7.37 | 4 | 46 | 0.12 | Fixed |
|  | PFS | 3 | 115 | 119 | 234 | 0.50 [0.35, 0.69] | 0.0001^***^ | 0.78 | 2 | 0 | 0.68 | Fixed |
|  | OS | 6 | 213 | 218 | 431 | 0.50 [0.33, 0.77] | 0.001 | 11.9 | 5 | 58 | 0.04 | Random |
| CIK treatments | | | | | | | | | | | | |
| only CIK cells applied | RFS | 6 | 457 | 407 | 864 | 0.58 [0.48, 0.70] | 0.00001^****^ | 5.86 | 6 | 0 | 0.44 | Fixed |
|  | PFS | 2 | 138 | 140 | 278 | 0.54 [0.41, 0.73] | 0.0001^***^ | 1.06 | 3 | 0 | 0.79 | Fixed |
|  | OS | 7 | 550 | 507 | 1057 | 0.62 [0.47, 0.82] | 0.0009 | 17.5 | 9 | 49 | 0.04 | Random |
| CIK cells + other lymphocytes | OS | 3 | 90 | 93 | 183 | 0.53 [0.34, 0.82] | 0.005 | 4.98 | 2 | 60 | 0.08 | Fixed |
| ≤1.5*10^10^ cells/cycle | RFS | 4 | 289 | 284 | 573 | 0.49 [0.25, 0.97] | 0.04 | 11.8 | 3 | 75 | 0.008 | Random |
|  | PFS | 2 | 96 | 98 | 194 | 0.51 [0.36, 0.71] | 0.0001^***^ | 1.24 | 3 | 0 | 0.74 | Fixed |
|  | OS | 5 | 361 | 362 | 723 | 0.51 [0.38, 0.69] | 0.00001^****^ | 7.85 | 6 | 24 | 0.25 | Fixed |
| ＞1.5*10^10^ cells/cycle | RFS | 2 | 160 | 117 | 277 | 0.51 [0.39, 0.68] | 0.00001^****^ | 1.1 | 2 | 0 | 0.58 | Fixed |
|  | OS | 4 | 241 | 200 | 441 | 0.61 [0.39, 0.97] | 0.04 | 11.5 | 4 | 65 | 0.02 | Random |
| ≤5 times of injection | RFS | 3 | 217 | 217 | 434 | 0.63 [0.47, 0.83] | 0.001 | 4.61 | 2 | 57 | 0.1 | Fixed |
|  | PFS | 2 | 138 | 140 | 278 | 0.54 [0.41, 0.73] | 0.0001^***^ | 1.06 | 3 | 0 | 0.79 | Fixed |
|  | OS | 6 | 364 | 366 | 730 | 0.54 [0.42, 0.69] | 0.00001^****^ | 14.2 | 7 | 51 | 0.05 | Fixed |
| ＞5 times of injection | RFS | 5 | 270 | 265 | 535 | 0.49 [0.38, 0.63] | 0.00001^****^ | 7.02 | 4 | 43 | 0.14 | Fixed |
|  | OS | 4 | 236 | 237 | 473 | 0.80 [0.60, 1.07] | 0.13 | 5.01 | 3 | 40 | 0.17 | Fixed |

*: *p*＜0.01; **: *p*＜0.001; ***: *p*＜0.0001，****: *p*＜0.00001.

CIK: cytokine-induced killer cells; TNM: Tumor/regional lymph node/metastasis stage system; BCLC: Barcelona Clinic Liver Cancer stage system; AJCC: American Joint Committee on Cancer staging system; PVT: portal venous thrombus.
