# Supplementary material for: Autologous transplantation of cytokine-induced killer cells as an adjuvant therapy for hepatocellular carcinoma in Asia: an update meta-analysis and systematic review
Source: Oncotarget. 2017 Feb 17;8(19):31318–28. doi: 10.18632/oncotarget.15454 (PMC5458210; doi:10.18632/oncotarget.15454)
Supplement: Supplementary file 6 [file oncotarget-08-31318-s006.docx]

**Finally included articles: n=12**

**Reference of the excluded studies after full-text screened.**

PubMed(116) EmBase(70) Cochrane(52) Web of Science(72)

**Title/abstract review: n=310**

Excluded studies: n = 19

-Repeating reports: n = 2 [[1](#_ENREF_1), [2](#_ENREF_2)]

-Unavailable data: n = 3 [[3-5](#_ENREF_3)]

-Non-comparative studies: n=2 [[6](#_ENREF_6), [7](#_ENREF_7)]

-retrospective studies: n=9 [[8-16](#_ENREF_8)]

-metastatic HCC: n=1 [[17](#_ENREF_17)]

-patients had received other anti-cancer therapy: n=2 [[18](#_ENREF_18), [19](#_ENREF_19)]

Full-text article screened(n=31)

Excluded studies: n =279

-Duplications: n = 69

-Irrelevant topics: n =98

-Non-comparative studies: n = 22

-Case-reports or reviews: n= 41

-Animal models or cell line: n= 49

1. Pan CC, Huang ZL, Li W, Zhao M, Zhou QM, Xia JC and Wu PH. Serum alpha-fetoprotein measurement in predicting clinical outcome related to autologous cytokine-induced killer cells in patients with hepatocellular carcinoma undergone minimally invasive therapy. Chinese Journal of Cancer. 2010; 29(6):596-602.

2. Cui J, Li W, Wang N, Zhao H, Jin H, Niu C and Wang G. Risk of HCC recurrence with cellular immunotherapy following radiofrequency ablation. Journal of Clinical Oncology. 2013; 31(15).

3. Xu YM, Zhang NZ, Zhang GL, Ma WQ, Gao CJ, Xu WD and Chen FX. Transcatheter hepatic arterial chemoembolization and percutaneous ethanol injection combined with cytokine-induced killer cells in treatment of advanced hepatocellular carcinoma. World Chinese Journal of Digestology. 2004; 12(6):1288-1291.

4. Liu L, Zhong JM, Chen XX, Ding N, Qian QJ and Qu ZQ. (2015). Transarterial chemoembolization combined with autologous DC-CIK cells for the treatment of hepatocellular carcinoma of BCLC C-stage: A randomized controlled study. [Chinese]. Journal of Interventional Radiology (China), pp. 434-438.

5. Liu ZZ, Gu J, Han T, Guo F, Li QH, Sun QQ, Guo X, Zheng ZD and Xie XD. Effect of sorafenib combined with CIK cell treatment on immunity and adverse events in patients with late-stage hepatocellular carcinoma. International Journal of Clinical and Experimental Medicine. 2016; 9(2):4625-4629.

6. Wang XP, Xu M, Gao HF, Zhao JF and Xu KC. Intraperitoneal perfusion of cytokine-induced killer cells with local hyperthermia for advanced hepatocellular carcinoma. World Journal of Gastroenterology. 2013; 19(19):2956-2962.

7. Zhang Z, Wang L, Luo Z, Zhao X, Huang J, Li H, Yang S, Zhao X, Zhang L, Li L, Wang F, Huang L and Zhang Y. Efficacy and safety of cord blood-derived cytokine-induced killer cells in treatment of patients with malignancies. Cytotherapy. 2015; 17(8):1130-1138.

8. Yang M, Guo Z, Si T, Xing W, Liu F, Li B, Yu H, 郭志, 司同国, 邢文阁, 刘方, 李保国 and 于海鹏. Influence of transcatheter arterial chemoembolization combined with cytokine-induced killer cell on the immune function in patients with advanced primary hepatocellular carcinoma. Journal of Interventional Radiology. 2011; 20(2):116-119.

9. Huang ZM, Li W, Li S, Gao F, Zhou QM, Wu FM, He N, Pan CC, Xia JC, Wu PH and Zhao M. Cytokine-induced killer cells in combination with transcatheter arterial chemoembolization and radiofrequency ablation for hepatocellular carcinoma patients. Journal of Immunotherapy. 2013; 36(5):287-293.

10. Pan K, Li Y-Q, Wang W, Xu L, Zhang Y-J, Zheng H-X, Zhao J-J, Qiu H-J, Weng D-S, Li J-J, Wang Q-J, Huang L-X, He J, Chen S-P, Ke M-L, Wu P-H, et al. The Efficacy of Cytokine-Induced Killer Cell Infusion as an Adjuvant Therapy for Postoperative Hepatocellular Carcinoma Patients. Annals of Surgical Oncology. 2013; 20(13):4305-4311.

11. Guo W, Liu L, Wu D, 刘莉 and 吴德华. Dendritic cell-cytokine induced killer cell immunotherapy combined with transcatheter arte-rial chemoembolization for hepatocellular carcinoma:safety and efficacy. Journal of Southern Medical University. 2014; 34(5):674-678.

12. Kou X, Huang Y, Yuan Z, Wu M and Qian Q. Efficacy of the combined therapy with autologous dendritic cells-cytokine induced killer cells and percutaneous microwave coagulation for hepatocellular carcinoma. Chinese Journal of Cancer Biotherapy. 2015; 22(4):509-513.

13. Pan QZ, Wang QJ, Dan JQ, Pan K, Li YQ, Zhang YJ, Zhao JJ, Weng DS, Tang Y, Huang LX, He J, Chen SP, Ke ML, Chen MS, Wicha MS, Chang AE, et al. A nomogram for predicting the benefit of adjuvant cytokine-induced killer cell immunotherapy in patients with hepatocellular carcinoma. Sci Rep. 2015; 5:9202.

14. Yang S, Zhao X, Huang J, Li H, Yan Y, Dong W, Huang L and Zhang Y. Anti-tumor effect of autologous cytokineinduced killer (CIK) cells in patients with advanced primary hepotic carcinoma. Cytotherapy. 2015; 17(6):S20-S21.

15. Chen JL, Lao XM, Lin XJ, Xu L, Cui BK, Wang J, Lin GH, Shuang ZY, Mao YZ, Huang X, Yun JP, Jin JT and Li SP. Adjuvant Cytokine-Induced Killer Cell Therapy Improves Disease-Free and Overall Survival in Solitary and Nonmicrovascular Invasive Hepatocellular Carcinoma After Curative Resection. Medicine. 2016; 95(5):e2665.

16. Qian L, Wang NY, Tian HM, Jin HF, Zhao HJ, Niu C, He H, Ge TW, Han W, Hu JF, Li D, Han FJ, Xu JT, Ding X, Chen JT, Li W, et al. Dual Effects of Cellular Immunotherapy in Inhibition of Virus Replication and Prolongation of Survival in HCV-Positive Hepatocellular Carcinoma Patients. Journal of Immunology Research. 2016.

17. Niu LZ, Li JL, Zeng JY, Mu F, Liao MT, Yao F, Li L, Liu CY, Chen JB, Zuo JS and Xu KC. Combination Treatment with Comprehensive Cryoablation and Immunotherapy in Metastatic Hepatocellular Cancer. European Journal of Cancer. 2014; 50:E29-E29.

18. Li W, Wang YM, Kellner DB, Zhao LD, Xu LP and Gao QL. Efficacy of RetroNectin-activated cytokine-induced killer cell therapy in the treatment of advanced hepatocelluar carcinoma. Oncology Letters. 2016; 12(1):707-714.

19. Long SQ, Zhang XX, Yang XB, Zhou YS, He WF, Liao GY, Ouyang YS, Li QP, Huang JP, Deng H, Pan ZQ, Xiao SJ, Cai JZ and Wu WY. [Combination of Jianpi Liqi Yiliu Formula with Cytokine-induced Killer Cell Treatment for Advanced Hepatocellular Carcinoma]. Zhongguo Zhong xi yi jie he za zhi Zhongguo Zhongxiyi jiehe zazhi = Chinese journal of integrated traditional and Western medicine / Zhongguo Zhong xi yi jie he xue hui, Zhongguo Zhong yi yan jiu yuan zhu ban. 2016; 36(2):160-165.
