# Supplementary material for: Autologous transplantation of cytokine-induced killer cells as an adjuvant therapy for hepatocellular carcinoma in Asia: an update meta-analysis and systematic review
Source: Oncotarget. 2017 Feb 17;8(19):31318–28. doi: 10.18632/oncotarget.15454 (PMC5458210; doi:10.18632/oncotarget.15454)
Supplement: Supplementary file 7 [file oncotarget-08-31318-s007.docx]

**Search Strategies**

**PubMed**

(cytokine induced killer cell) AND "Carcinoma, Hepatocellular/therapy"[Majr]

Results: 116

**Embase**

("cytokine induced killer cell*" OR "cytokine-induced killer cell*" OR "CIK cell*" OR "lymphocyte-activated killer cell*" OR "lymphocyte activated killer cell*") AND ("hepatocellular carcinoma*" OR "liver cell carcinoma" OR "liver cancer" OR hepatoma OR "liver neoplasm*")

Results:70

**The Cochrane Library**

"hepatocellular carcinoma" AND "cytokine induced killer cell*" OR "CIK cell*" OR "lymphocyte-activated killer cell*" OR "lymphocyte activated killer cell*" use filter of “trials”

Results:52

**Web of Science**

TS=("cytokine induced killer cell*" OR "cytokine-induced killer cell*" OR "CIK cell*") AND TS=("hepatocellular carcinoma*" OR "liver cell carcinoma" OR "liver cancer" OR hepatoma OR "liver neoplasm*")

Results:72
